# The Relationship between Diaspore Characteristics with Phylogeny, Life History Traits, and Their Ecological Adaptation of 150 Species from the Cold Desert of Northwest China

**DOI:** 10.1155/2014/510343

**Published:** 2014-01-30

**Authors:** Hui-Liang Liu, Dao-Yuan Zhang, Shi-Min Duan, Xi-Yong Wang, Ming-Fang Song

**Affiliations:** ^1^Key Laboratory of Biogeography and Bioresource in Arid Land, Xinjiang Institute of Ecology and Geography, Chinese Academy of Sciences, Urumqi 830011, China; ^2^Turpan Eremophytes Botanical Garden, Chinese Academy of Sciences, Turpan 838008, China

## Abstract

Diaspore characteristics of 22 families, including 102 genera and 150 species (55 represented by seeds and 95 by fruits) from the Gurbantunggut Desert were analyzed for diaspore biological characteristics (mass, shape, color, and appendage type). The diaspore mass and shape were significantly different in phylogeny group (APG) and dispersal syndromes; vegetative periods significantly affected diaspore mass, but not diaspore shape; and ecotypes did not significantly affect diaspore mass and shape, but xerophyte species had larger diaspore mass than mesophyte species. Unique stepwise ANOVA results showed that variance in diaspore mass and shape among these 150 species was largely dependent upon phylogeny and dispersal syndromes. Therefore, it was suggested that phylogeny may constrain diaspore mass, and as dispersal syndromes may be related to phylogeny, they also constrained diaspore mass and shape. Diaspores of 85 species (56.67%) had appendages, including 26 with wings/bracts, 18 with pappus/hair, 14 with hooks/spines, 10 with awns, and 17 with other types of appendages. Different traits (mass, shape, color, appendage, and dispersal syndromes) of diaspore decided plants forming different adapted strategies in the desert. In summary, the diaspore characteristics were closely related with phylogeny, vegetative periods, dispersal syndromes, and ecotype, and these characteristics allowed the plants to adapt to extreme desert environments.

## 1. Introduction

Heritable characteristics of seeds that contribute to seed and seedling survivorship under natural conditions are open to natural selection. Sexual reproduction can improve the success rate of breeding more than asexual reproduction for plants in the face of adversity, so in response to plant propagation, sexual reproduction is the focus of the study [1]. Seeds are a component of such a set; flower and fruit type, the type of placentation, the number of ovules per ovary, and the process of embryo development are traits that are generally evolutionarily conservative and strongly associated with family membership and seed mass [2]. Natural selection that maintains phenotypic constancy in these traits may preclude evolutionary change in seed mass if it is developmentally and genetically correlated with them. In any case, the strong taxonomic effect on seed mass suggests that there are factors other than the ecological features measured in this study that determine seed mass [3]. Diaspore mass and shape is a core characteristic in the life history of a plant [4]. Variation of the diaspores between or within species has important ecological and evolutionary significance [5]. Characteristics of diaspore can be used as an important basis for taxonomy. Many previous studies have shown that the type of plant diaspores and their morphological characteristics, such as mass, shape, color, and appendages, as well as fecundity pattern and postdispersal level, are closely related to their life-form, dispersal syndrome, reproductive strategy, seed germination, seedling settlement, and population distribution, in which seed mass and shape were effective in dispersal syndromes, dispersal distance, and longevity of the soil seed bank [6–9].

A comparative study based on a large sample will enable ecologists to distinguish the main ways plants adapt to evolution and identify the plants with fitness (or lack of fitness) showing the physiological characteristics of life history in specific habitats [10]. Currently a study on a large sample of the diaspore characteristics in a same floristic has become a research hotspot of ecology, such as tropical wetlands in Venezuela [3, 6, 11], various habitats in Europe [12], New Zealand forests, and semiarid areas of Australia [7–9, 13], while the mainly focuses on the Inner Mongolia grassland and Horqin sandy in China [14–16] and the Qinghai-Tibet plateau alpine meadow communities [17, 18]. However, less information is available regarding on diaspore traits in the arid cold desert area in northwest China, but referred seed dispersal traits of 24 cruciferous short-lived plants [19].

Information on seed dispersal of desert plants is crucial in order to understand adaptative strategies of plants in desert areas. Our aim in this study is to discuss (1) the relationship of biological characteristics with phylogeny group (APG), vegetative periods, dispersal syndromes, and ecotypes and (2) the relationship between biological characteristics and dispersal adaptation to the desert ecological environment. The study may utilize to further reveal the universal pattern of plant life history and reproductive strategies in this cold desert and ulteriorly understand the continuous mechanisms for desert vegetation, population-proliferation regime, weed invasion mechanisms, and biodiversity loss mechanisms. Therefore, it has a great significance in taxonomy, ecology, and evolutionary biology for studying other cold deserts.

## 2. Materials and Methods

### 2.1. Study Area and Species Traits

The cold desert is well-known due to it being located in colder areas with and higher latitude; and it is a dry, cold area of land that receives almost no precipitation. When it does, it is usually in the form of snow or fog [20]. The Gurbantunggut Desert ranged in latitude from 44°11′–46°20′ and longitude from 84°31′–90°00′, with an area of 4.88 × 10^4^ km^2^; it is the second largest desert in China. It does not only contain the largest fixed and semifixed desert in the central region but also contains a salination desert in the southern edge, so it formed an abundant xerophytes and halophytes community [21]. This area is a typical inland temperate desert climate. In this area, the mean annual temperature is 7.3°C and the winter temperature could fall down to −20°C. The annual rainfall is very low in the summer, but there is significant snow in winter and spring (the largest number of snow thickness is between 20 and 30 cm) [22]. The stable wet sand layer by melting snow provides an important guarantee for plants survival and formation, so the species richness is relatively higher in this desert than other central deserts richness is relatively higher including 206 species [21]. Therefore, plant types with both short and long vegetative periods evolved. The natural vegetation in the desert is dominated by *Haloxylon ammodendron* and *Haloxylon persicum* [21]. Herbaceous plants are widespread and abundant in spring and early summer. Short-lived or ephemeral plants obtain certain development. Amaranthaceae is in a clearly dominant position while Brassicaceae, Asteraceae, Fabaceae, Poaceae, and so forth are common [21, 23, 24].

### 2.2. Composition of Materials

In this paper, 150 plant species were selected for the study and classified into 28 families and 102 genera, which accounted for 72.8% of species, 82.9% of genera, and 93.3% of family in this area. Among them, there was one gymnosperm (0.67%), 15 monocotyledon (10.00%), with dominant Poaceae (13 species, 8.67%), and 134 dicotyledon (89.33%), with dominant Amaranthaceae (38 species, 25.33%), Brassicaceae (20 species, 13.33%), and Asteraceae (14 species, 9.33%). They were divided into 10 APG II taxonomic phylogeny groups as follows [25]: Coniferopsida, Monocots, Commelinids, Eudicots, Core eudicots, Rosids, Eurosids I, Eurosids II, Euasterids I, and Euasterids II (Table 1).

Plant types with both short and long vegetative periods were evolved in this area [24] and short (ephemeral) plants included annuals, ainnuals/biennials, and biennials herb, so vegetative periods were divided into annuals (AH), annuals/biennials (ABH), biennials (BH), biennials/perennials (BPH), perennials (PH), shrubs (S), semishrubs (SS), small arbor (SA), annuals ephemerals (AE), annuals/biennials ephemerals (ABE), and biennial ephemerals (BE) (Table 1).

Ecotypes were divided into 2 categories: xerophyte (67 species, 44.67%) and mesophyte (83 species, 55.33%) (Table 1).

### 2.3. Study Methods on Morphology Characteristics and Dispersal Syndromes

#### 2.3.1. Morphology Characteristics

Metrical objects of 150 species could be divided into seeds (55 species) and fruits (95 species), which could be further divided into various types.

1. Mass: With reference to Thompson's method [26], we randomly selected 100 seeds or fruits in each species, measuring the weight (g) with fine balance (Sartorius BS110S, accuracy to 0.0001 g). Each species had five repeats, and then we took the average value and calculated the standard error. If the appendages were valuable for dispersal, we measured including them.
2. Shape: according to Thompson et al.'s methods [26], the seed shape was calculated as the variance of the three main perpendicular dimensions after dividing all values by length. Totally spherical seeds would have shape = 0, with this value increasing as they became flatter or elongated. In other words, larger values of variance were associated with flatter seeds; smaller variance indicated more round seeds. According to the three-dimensional mean variance, we classified them into seven grades and calculated the frequency of occurrence (percentage) at each grade. Finally, combining observation and [6, 23], we determined the shape of each species and calculated the frequency (percentage) of each shape group.
3. Color: combining observation and [6, 23], we can determine the diaspore color of each species and calculate the frequency (percentage) of each color group.
4. Appendage: We observed and recorded the appendage features, such as wing/bract, pappus/hair, hook/spine, awn, or other kinds of appendages (such as style/perianth/beak/warts/placenta, etc.).

#### 2.3.2. Dispersal Syndromes

Because seed dispersal was divided into two phases. (1) Phase I dispersal represents the movement of the seeds from the parent plant to a surface, each of 150 study species were assigned to one of five dispersal syndromes in their primal dispersal phase, on the basis of data from field collections, observing seed ornamentation and appendages and descriptions from published flora [27–29]. *①* Zoochorous species are defined as having awns, spines, or hooks to adhere to animals (epizoochory) or seed with fleshly or arillate fruits for animals to eat (endochory); *②* anemochorous species are defined as having membranous wings, bracts, perianth, balloon, hair, or dust seed (<0.01 mg); *③* autochorous species are divided into ballistically dispersed species possessing explosively dehiscing capsules by wind and by wetting that throw the seeds some distance from the parent plant; *④* barochorous species are defined as those lacking any obvious dispersal mechanism or disperser reward; and *⑤* ombrohydrochory dispersed species are defined as the seeds producing a mucilage upon being wetted (Table 1). (2) Phase II dispersal includes both horizontal and vertical movement of the seeds after arrival on the surface until it is lodged or germinated. Though many species are subject to secondary dispersal by animals (major ants (myrmecochory), indicated by the presence of an elaiosome or an appendage on seeds that is attractive to ants) or water, for the purpose of this analysis it was examined only the primary phase of dispersal. Meanwhile, ants as a mass of predators, the seeds were divided to secondary dispersal type.

### 2.4. Data Analysis

SPSS 15.0 was used for calculating the mean and the standard error of data. The ANOVA method (SPSS 15.0) was applied to analyze the significant difference between the diaspore mass (weight)/shape in different APG, vegetative periods, ecotypes, and dispersal syndromes. To examine differences in diaspore mass and shape among vegetative periods and taxonomic class rank, we used the Kruskal-Wallis test (*K*-*W*) after categorization of the variables. The association between nominal traits was determined with the Pearson *x* ^2^ test-statistic. Correlations between quantitative traits were examined using Pearson correlation coefficient. Diaspore mass was log-transformed prior to statistical analysis. One-way analysis of variance ANOVA was applied after verifying the homogeneity of variance by Levene's test.

## 3. Results

### 3.1. The Relationship between Diaspore Mass with Phylogeny, Life History Traits, and Ecotype

**Figure 1:**
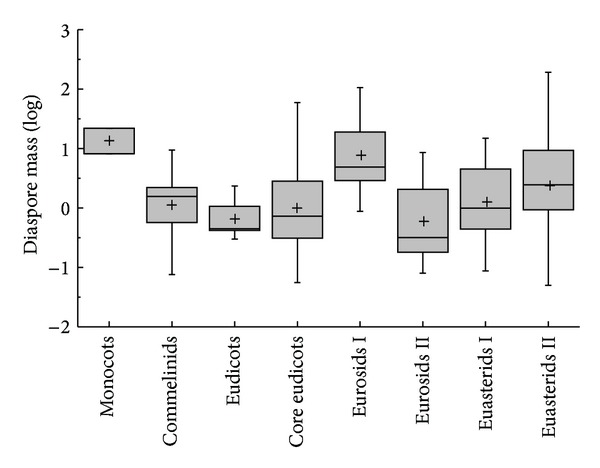
Box plots showing mean (+), median (—), quartiles, and outliers (-) of diaspore mass of 150 species grouped by different APG II taxonomic phylogeny group. Because Coniferopsida and Rosids are only one species, they do not compare with others.

**Figure 2:**
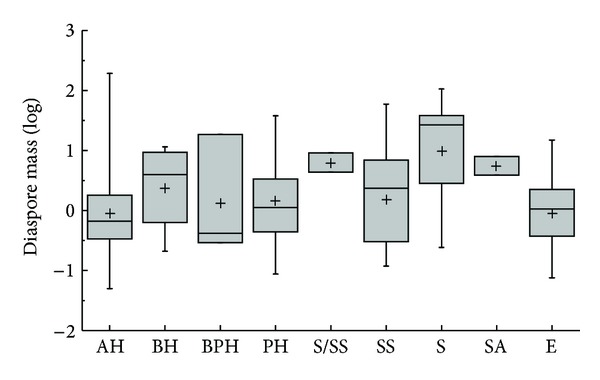
Box plots showing mean (+), median (—), quartiles, and outliers (-) of diaspore mass of 150 species grouped by different vegetative periods. Because annual-biennial (ABH) species is only one species, it does not compare with others. AH = annuals; ABH = annuals/biennials; BH = biennials; BPH = biennials/perennials; PH = perennials; S = shrubs; SS = semishrubs; SA = small arbor; AE = annuals ephemerals; ABE = annuals/biennials ephemerals; BE = biennial ephemerals.

**Figure 3:**
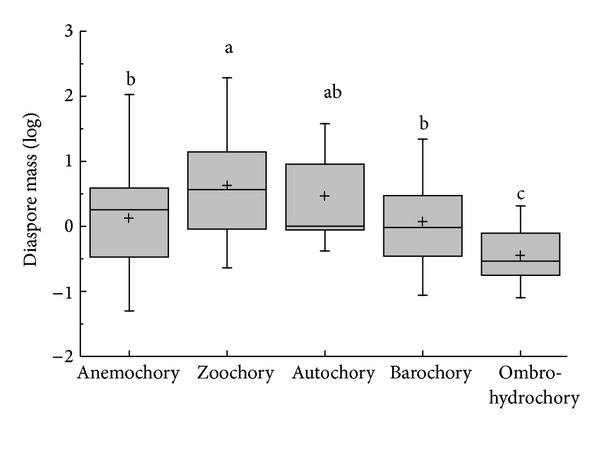
Box plots showing mean (+), median (—), quartiles, and outliers (-) of diaspore mass of 150 species grouped by different dispersal syndromes.

**Figure 4:**
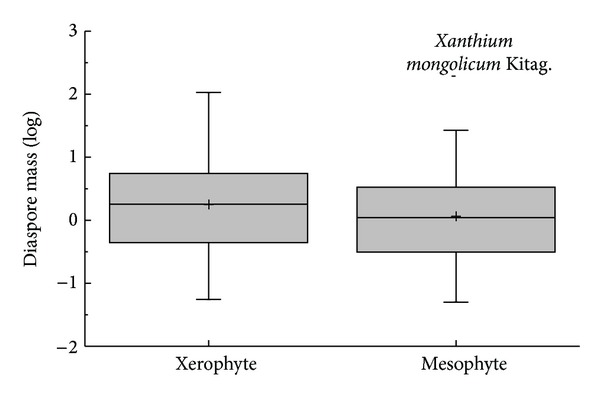
Box plots showing mean (+), median (—), quartiles, and outliers (-) of diaspore mass of 150 species grouped by different ecotypes

The species of Core eudiocot were the most abundant in all groups of APG II (Figure 1). Except for Coniferopsida and Rosids, which are only one species in their APG II taxonomic group, diaspore mass differed significantly among the phylogenic group (*K*-*W*: *H* = 29.938, df = 7, *P* < 0.001); group of Monocots had the highest diaspore mass; and Eudicots had the smallest diaspore mass (Figure 1). Except for ABH, the diaspore mass differed significantly among the vegetative period (*K*-*W*: *H* = 17.677, df = 8, *P* = 0.024); the species of shrub had the highest diaspore mass and ephemerals had the smallest diaspore mass (Figure 2). The diaspore mass differed significantly among dispersal syndromes (*F* = 8.383, df = 4, *P* < 0.001); the zoochorous species had the highest diaspore mass and the ombro-hydrychorous species had the smallest diaspore mass (Figure 3). The dispersal syndromes had a significant relationship with APG (*K-W*: *H* = 75.921, df = 7, *P* < 0.001) and vegetative period (*K-W*: *H* = 28.108, df = 8, *P* < 0.001). The second dispersal seeds by ants were about 72.7% (Table 1). There were significant differences between diaspores mass and ant dispersal (*Z* = −3.343, *P* = 0.001). The diaspore mass did not differ significantly among ecotypes (*Z* = −1.701, *P* = 0.089), but the species of xerophytes had a higher diaspore mass than the species of mesophyte (Figure 4).

### 3.2. The Relationship between Diaspore Shape with Phylogeny, Life History Traits, and Ecotype

**Figure 5:**
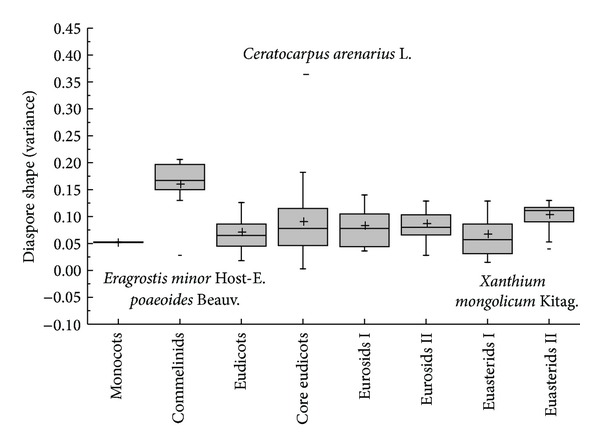
Box plots showing mean (+), median (—), quartiles, and outliers (-) of diaspore shape (variance) of 150 species grouped by different APG II taxonomic phylogeny group. Because Coniferopsida and Rosids are only one species, they do not compare with others.

**Figure 6:**
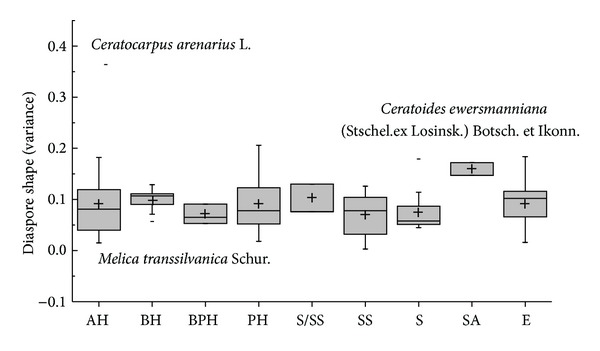
Box plots showing mean (+), median (—), quartiles, and outliers (-) of diaspore shape (variance) of 150 species grouped by different vegetative periods. Because annual-biennial (ABH) species is only one species, it does not compare with others. AH = annuals; ABH = annuals/biennials; BH = biennials; BPH = biennials/perennials; PH = perennials; S = shrubs; SS = semishrubs; SA = small arbor; AE = annuals ephemerals; ABE = annuals/biennials ephemerals; BE = biennial ephemerals.

**Figure 7:**
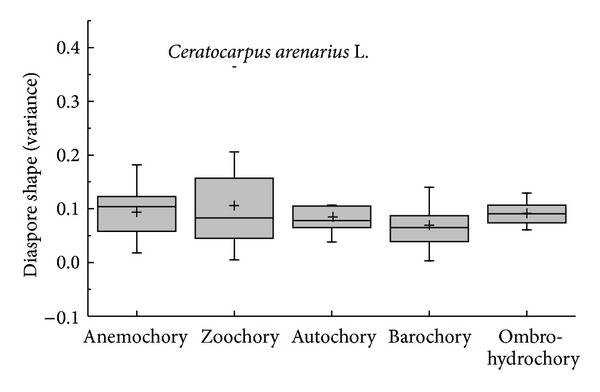
Box plots showing mean (+), median (—), quartiles, and outliers (-) of diaspore shape (variance) of 150 species grouped by different dispersal syndromes.

**Figure 8:**
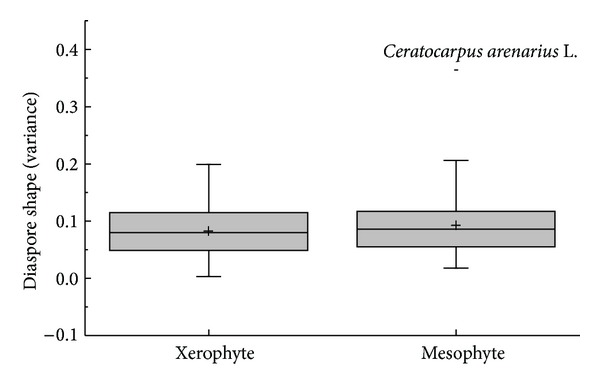
Box plots showing mean (+), median (—), quartiles, and outliers (-) of diaspore shape (variance) of 150 species grouped by different ecotypes.

**Figure 9:**
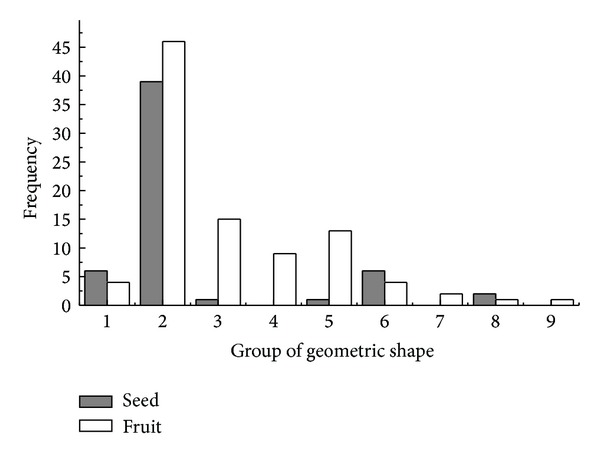
Frequency distribution of diaspore geometric shape. (1) Spheroideus, nearly-spheroideus; (2) elipsoid, broad-elipsoid, narrow-ellipsoid, ovoid, elongated-ovoid, obovoid, elongated-obovoid, subulate-ovoid, cylindrical-obovoid, spherical, spherical-ovoid; (3) lenticular, planular-ovoid, oblate-disc; (4) cylindrical, conical; (5) spindly, lanceolate, needle, Linearis; (6) reni; (7) arcuatus, curved; (8) trigonous, triqueter; (9) fan, rhombus.

The diaspore mean shape variance showed differences in APG II group (*K-W*: *H* = 29.120, df = 7, *P* < 0.001) and dispersal syndromes (*F* = 3.596, df = 4, *P* = 0.008); the species of Commelinids of zoochorous had the largest shape mean variance and the species of Monocots and ombro-hydrychory had smallest shape mean variance (Figures 5 and 7), but not in different vegetative period (*K-W*: *H* = 9.101, df = 8, *P* = 0.334) (Figure 6) or ecotype (*Z* = −0.830, *P* = 0.407) (Figure 8). According to shape mean variance and observation, the diaspore shape of 150 species could be divided into the following nine groups (Figure 9), of which 63.33% (95 species) are close to spherical or oval. There were significant differences between diaspore shape and ant dispersal (*Z* = −2.218, *P* = 0.027). Two ANOVAs showed only significant interaction between APG and dispersal syndromes in shape variance (*F* = 2.707, *P* = 0.003).

The the source of variance is following by Mazer's method [3], multi-ANOVAs detected that variance in the diaspore mass accounted for 17.2% by APG, 6.6% by vegetative period, 16.1% by dispersal syndromes, and 0.1% by ecotype, while in the diaspore shape it was accounted 12.9% by APG, 5.5% by vegetative period, 3.9% by dispersal syndromes, and 0.2% by ecotype (Table 2).

### 3.3. Diaspore Color

**Figure 10:**
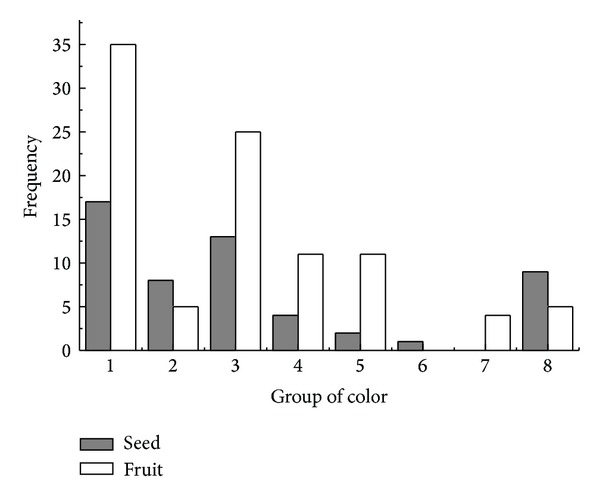
Frequency distribution of diaspore color. (1) Light brown, brown, dark brown, nut-brown; (2) light reddish brown, reddish brown, dark reddish brown; (3) light yellowish brown, yellowish brown, dark yellowish brown; (4) pale yellow, yellow, orange, reddish yellow; (5) light green, green, dark green, brownish green, yellowish green; (6) white; (7) grey, greyish white, greyish black; (8) black.

According to comparison and observation, the diaspore color of 150 species could be divided into the following eight groups (Figure 10), of which 68.67% (103 species) are close to brown. Diaspore color and ant dispersal had no significant relationship (*Z* = −1.109, *P* = 0.267).

### 3.4. Diaspore Appendages

Of the 150 species examined, 85 species (56.67%) had typical appendages, in which (1) 26 species (17.33%) had wings or bracts, which effectively spread with the wind; (2) 18 species (12.00%) had pappus or hairs, which effectively spread with the wind or stuck on animals; (3) 14 species (9.33%) had hooks or spines, which effectively hook on animals; (4) 10 species (6.67%) had awns, which effectively hang on animals or insert into the soil cracks for colonization; and (5) 17 species (11.33%) had other appendages, including style, perianth, beak, warts, placenta, and so forth, separately helping in different ways of dispersal (Table 1). The diaspores with appendage have a significant relationship with diaspore mass (*Z* = −4.508, *P* < 0.001) and diaspore shape (*Z* = −2.682, *P* = 0.007). This indicated that diaspores with appendage trended to large diaspore mass and irregular shape.

## 4. Discussion

### 4.1. Comparison of Diaspore Mass and Shape among APG, Vegetative Periods, Ecotypes, and Dispersal Syndromes

Diaspore mass might be the result of both selective pressures over a long-term ecological process and the constraints over the long-standing evolutionary history of the taxonomic group. Phylogenetic effects on life history traits have been interpreted as “phylogenetic constraints,” defined as “properties shared by the members of a monophyletic group by virtue of their common ancestry, which limits the response of these taxon to directional selection” [1]. Similarly, Moles et al. [30, 31] used phylogenetic analyses to infer the evolution of seed size for ca. 13 000 plant species and found that despite wide divergences in seed size, there was evidence of phylogenetic constraints on this trait. In this paper, diaspore mass and shape showed significant differences (*P* < 0.05) among APG groups, indicating that the phylogenetic factor was one of the prerequisites for adaptation. This may suggest that phylogeny imposes limits to variability in reproductive traits within a clade, because of similar developmental and design constrains in related species. Miles and Dunham [32] also pointed out that any comparative study lacking a phylogenetic perspective would be incomplete.

Vegetative periods of plants have a close relationship with adaptation to interference [33]. In this paper, diaspore mass showed significant differences (*P* < 0.05) among vegetative periods in general, while the variance in shape did not show much difference among vegetative periods. It was indicated that diaspore mass was more effective than diaspore shape in seed dispersal between different vegetative periods in this area.

Diaspore mass and shape are also related to vegetation dynamics [33]. Diaspore mass and shape showed no significant differences (*P* > 0.05) among ecotypes overall, but the species of xerophyte had a far greater average mass than mesophyte, indicating that xerophyte plants often increased diaspore mass to reduce the displacement and increase the probability of effective colonization. Harel et al. [34] found that seed mass significantly decreased with increasing aridity and rainfall variability in seven out of fifteen in the hot desert of Israel. Butler et al. [33] reported that seed diameter and size in high-rainfall sites trended to have smaller seeds in the rain forest of Australia. Thus, we inferred that diaspore mass might be related with the rainfall or moisture in different ecosystems; in other words, plants in the drier environments produced larger diaspore mass.

Diaspore mass and shape showed significant differences among dispersal syndromes, which indicated that both of them were key factors in determining the dispersal syndrome. Moles et al. [35] had investigated a total of 11481 species from 10 vegetation type categories and found that in 40–50 latitude zone, seeds trend to wind dispersal, but this data is absent in the cold desert. In this paper, diaspores of 45 species were light and round shape (single mass less than 1 mg and three-dimensional mean variance less than 0.090), in which there were 21 species (46.67%) as annual herbaceous or ephemeral plants, tending to take the wind for large-scale dispersal, while the heavy or irregularly shaped (often as a result of the existence of appendage) fruits often disperse in virtue of animals or self-transmission [4, 6]. Our data proves this theory could be expanded in this cold desert. In addition, Thomson et al. [36] used generalized linear mixed models with basic life-history and ecological traits to predict seed dispersal mechanisms and found that actual dispersal mechanisms (c.50% correct) was equally well to inferred dispersal mechanisms by the model; whether this model is also suitable for this desert still needs to be examined in the future.

This phylogenetic pattern of diaspore mass was previously shown in different floras [37]. In this study, we synthesized information on phylogenetic, life history, and ecological factors, using unique stepwise ANOVAs to infer the correlations between diaspore mass/shape and phylogeny, life history, and ecotype. The result of this study showed that variance in diaspore mass and shape among these 150 species is largely dependent upon phylogeny and seed dispersal syndromes. Therefore, it was suggested that phylogeny may constrain diaspore mass, and as dispersal syndromes may be related to phylogeny, they also constrain diaspore mass and shape. That is, inherent characteristics of species may play a prominent role in evolution of diaspore mass and shape, and stochastic factors such as environmental conditions are also important selective pressures.

### 4.2. Diaspore Morphological Characteristics and Dispersal Syndrome Adaptative to the Desert Environment

Plants growing in the Gurbantunggut Desert developed relevant diaspore morphology characteristics and dispersal syndromes adaptative to the desert environment in the long-term evolution. The Gurbantunggut Desert had a typical arid climate, including deeply buried groundwater and lack of surface runoff; most survivors in this environment were xerophyte plants [21]. *Haloxylon persicum* community developed well at the top and upper section of sand dunes, accompanied by *Stipagrostis adscensionis*, *Stipagrostis pennata*, *Eremosparton songoricum,* and *Agriophyllum squarrosum*, and so forth. Therefore, plants growing on moving sand dunes often had middle (*Haloxylon persicum*) or large (*Eremosparton songoricum*) weighted diaspores. Some of them were slim shaped although light weight (*Stipagrostis adscensionis*, *Stipagrostis pennata*, *Corispermum lehmannianum*, etc.), being effective against long-distance dispersal and in occupying the surrounding optimizational environment [15]. On the other hand, there were extensive biological soil crusts at the bottom and lower section of sand dunes, which played an important role in sand-fixation [22]. Plants living here must develop their diaspores to adapt the uniform and dense “shell” [38, 39]; thus they were generally small and light or had appendages which enable them to effectively disperse by the wind, pass through the cracks of the biological soil crusts, and settle down, such as *Erodium oxyrrhynchum*, *Stipagrostis adscensionis*, and *S. pennata*, which could take a special way named “active drill” into soil cracks using awns or needles. The small diaspore of *Bassia dasyphylla*, *Bassia sedoides*, *Kochia iranica,* and *Camphorosma monspeliaca* had hooks/spines or short hairs, and enabled them to dispese via wind or animal Genus *Nitraria* had bright and juicy berries, which could attract animals feeding in order to improve wide-ranged dispersal. In contrast, most species of Fabaceae and Zygophyllaceae which had large and heavy diaspores, such as genus* Glycyrrhiza*, *Sophora alopecuroides*, and *Zygophyllum fabago*, mainly used to take full advantage of the favorable surrounding nutritional conditions. Thomson et al. [40] found that once a plant height was accounted for, the small-seeded species dispersed further than did large-seeded species. Our results were focusing only on diaspore mass and morphological characteristics, not taking into account plant height. In the future study, we will try to reveal whether small-seeded species may disperse further from the parent plant, accounting for plant height, than do large-seeded species in this desert?

There was a certain proportion of salt desert and salinity wasteland in Gurbantunggut Desert peripheral areas especially on the southern edge, where distributing a variety of typical halophytes or wide adaptable plants [21]. Among them, *Althaea officinalis*, *Dodartia orientalis*, *Peganum harmala,* and most species of Amaranthaceae had small and light diaspores (single dry weight less than 1 mg) and close to spherical (three-dimensional mean variance less than 0.090). They were not only easy to disperse by wind but also effective at forming persistent soil seed bank [8, 9, 13, 26]. Typical halophytes of genus* Atriplex*, *Anabasis*, *Halogeton,* and *Salsola* were usually wind-borne with the flat wing-like appendages, but when the rainfall was enough they could also drift on the water surface to a farther place.

Mesophyte was also an important part of the flora and a majority of them were weeds. Their diaspores were small, and light mass, they effectively improved the dispersal range and effective reproductive rate, such as *Heliotropium ellipticum*, *Eragrostis minor*, *Hyoscyamus niger,* and many species of Brassicaceae and Labiatae. Diaspores with appendages like wings/bracts or pappus/hairs were generally wind-borne and those with hooks/spines were easy to stick on animals for long-distance spread or insert into soil cracks to settle. Besides, diaspores of *Plantago lessingii*, mostly Brassicaceae and Labiatae mesophyte plants had mucilage which is an effective means to resist against environmental and man-made interference.

On the surface, the brown-color which was close to the sand color could help them to avoid been eaten by ants. However, it was found that the diaspore color and ant dispersal had no significant relationship (*Z* = −1.109, *P* = 0.267). It may suggest that the ant could not see the diaspore color; they looked for the food relying on the seed appendage or elaiosome. It was concluded that diaspore morphology characteristics and dispersal syndromes would cause some adaptive changes due to different settling environments.

In general, the diaspore characteristics were closely related to phylogeny, vegetative periods, dispersal syndromes and ecotype, and these characteristics allowed the plants to adapt extreme desert environments. Diaspore characteristics of plants in this area are influenced by natural selection forces. This study has provided new insights into diaspore characteristics and their ecological adaptation in this cold desert. However, there are still many unanswered questions concerning key aspects of the dispersal traits. These are key research questions arising from this study, and important ones that will need to be addressed in the future.

## Figures and Tables

**Table 1:** The species, APG II taxonomic phylogeny group, family, vegetative period, metrical object, diaspore characteristics (length, width, height, shape (variance), color, appendages), first dispersal phase (dispersal syndrome), second dispersal phase, and ecotype of 150 species in the Gurbantunggut Desert, northwest China.

APG II taxonomic phylogeny group	Family	Species	Vegetative period	Metrical object	Mass of 100 seeds (Mean ± SE)	Length, width, and height (Mean ± SE)	Diaspore shape variance	Diaspore color	Appendages	First dispersal phase (dispersal syndromes)	Second dispersal phase	Ecotype
Coniferopsida	Ephedraceae	*Ephedra przewalskii* Stapf	S	Cone	280.86 ± 7.06	3.926 ± 0.054 1.700 ± 0.051 0.854 ± 0.054	0.114	Light brown	Bract	Anemochory	Ant	Xerophyte

Monocots	Liliaceae	*Eremurus inderiensis* (M. Bieb.) Regel	PH	Seed	820.62 ± 5.85	5.766 ± 0.125 3.454 ± 0.089 2.788 ± 0.073	0.052	Brown	Wing	Anemochory	Ant	Xerophyte
	Iridaceae	*Iris lactea* Pall. var. *chinensis* (Fisch.) Koidz.	PH	Seed	2199.16 ± 30.34	4.320 ± 0.146 3.408 ± 0.107 2.002 ± 0.085	0.053	Reddish brown	None	Barochory	Ant	Mesophyte

Commelinids	Poaceae	*Achnatherum inebrians* (Hance) Keng	PH	Seed	90.50 ± 0.63	3.976 ± 0.071 0.704 ± 0.015 0.684 ± 0.015	0.157	Brown	Hair	Zoochory	Ant	Xerophyte
		*Stipagrostis adscensionis* L.	AH	Caryopsis	57.00 ± 1.15	17.64 ± 0.812 4.422 ± 0.491 2.636 ± 0.148	0.150	Brownish green	Awn	Zoochory	—	Xerophyte
		*Stipagrostis pennata* Trin.	PH	Caryopsis	88.16 ± 0.53	21.764 ± 0.890 4.214 ± 0.393 2.412 ± 0.240	0.167	Yellowish green	Awn	Zoochory	—	Xerophyte
		*Chloris virgata* Sw.	AH	Caryopsis	39.72 ± 0.80	1.850 ± 0.044 0.574 ± 0.028 0.378 ± 0.016	0.130	Pale yellow	Awn	Zoochory	Ant	Mesophyte
		*Eragrostis minor* Host-E. poaeoides Beauv.	AE	Seed	7.58 ± 0.24	0.584 ± 0.021 0.440 ± 0.017 0.372 ± 0.017	0.028	Reddish brown	None	Anemochory	—	Mesophyte
		*Eremopyrum bonaepartis* (Spreng.) Nevski	AE	Caryopsis	177.86 ± 4.59	9.570 ± 0.377 1.324 ± 0.043 1.042 ± 0.072	0.176	Yellowish green	Awn	Zoochory	Ant	Mesophyte
		*Eremopyrum triticeum* (Gaertn.) Nevski	AE	Caryopsis	184.76 ± 3.20	11.748 ± 0.482 1.394 ± 0.083 1.096 ± 0.041	0.184	Yellowish green	Awn	Zoochory	Ant	Xerophyte
		*Elymus atratus* Turcz.	PH	Caryopsis	155.82 ± 1.37	19.574 ± 1.02 1.394 ± 0.065 0.652 ± 0.034	0.206	Pale yellow	Awn	Zoochory	Ant	Mesophyte
		*Elymus sibiricus* L.	PH	Caryopsis	221.22 ± 4.19	15.372 ± 0.966 1.466 ± 0.066 0.638 ± 0.025	0.199	Light green	Awn	Zoochory	Ant	Xerophyte
		*Leymus racemosus* (Lam.) Tzvel.	PH	Caryopsis	944.44 ± 14.73	13.708 ± 0.872 2.736 ± 0.230 2.166 ± 0.157	0.156	Pale yellow	Awn	Zoochory	Ant	Mesophyte
		*Melica transsilvanica* Schur	PH	Caryopsis	42.60 ± 1.04	4.668 ± 0.112 2.116 ± 0.048 2.060 ± 0.037	0.071	Pale yellow	Hair	Zoochory	Ant	Mesophyte
		*Stipa capillata* L.	PH	Caryopsis	334.06 ± 5.20	10.758 ± 0.301 0.814 ± 0.039 0.788 ± 0.038	0.197	Pale yellow	Awn	Zoochory	Ant	Mesophyte
		*Stipa sareptana* Beck.	PH	Caryopsis	367.18 ± 2.76	12.512 ± 0.369 0.710 ± 0.028 0.676 ± 0.030	0.205	Yellowish brown	Awn	Zoochory	Ant	Mesophyte

Eudicots	Papaveraceae	*Corydalis stricta* Steph.	PH	Seed	44.66 ± 0.84	1.420 ± 0.043 1.268 ± 0.027 0.488 ± 0.016	0.086	Black	Placenta	Barochory	—	Xerophyte
		*Glaucium squamigerum* Kar. et Kir.	BPH	Seed	41.88 ± 0.18	1.272 ± 0.044 0.714 ± 0.022 0.542 ± 0.012	0.065	Black	None	Autochory	—	Xerophyte
		*Hypecoum erectum* L.	AE	Seed	29.96 ± 0.28	1.016 ± 0.038 0.822 ± 0.032 0.526 ± 0.020	0.045	Dark brown	Wart	Barochory	—	Xerophyte
	Ranunculaceae	*Ceratocephalus testiculatus* (Crantz) Bess.	AE	Achenecetum	106.46 ± 0.99	4.614 ± 0.229 4.548 ± 0.276 3.600 ± 0.180	0.018	Black	Beak/spine	Zoochory	—	Mesophyte
		*Clematis songarica* Bge.	SS	Achene	234.84 ± 11.31	4.038 ± 0.106 1.800 ± 0.044 0.654 ± 0.023	0.126	Brown	Pappus	Anemochory	—	Mesophyte

Core eudicots	Caryophyllaceae	*Gypsophila perfoliata* L.	PH	Seed	31.06 ± 0.45	0.944 ± 0.010 0.784 ± 0.023 0.460 ± 0.009	0.048	Black	Wart	Barochory	—	Mesophyte
	Amaranthaceae	*Agriophyllum squarrosum* (L.) Moq.	AH	Seed	115.82 ± 2.70	2.208 ± 0.093 1.712 ± 0.074 0.524 ± 0.040	0.108	White	None	Barochory	Ant	Xerophyte
		*Anabasis aphylla* L.	SS	Utricle	114.36 ± 1.98	2.064 ± 0.062 1.454 ± 0.061 0.496 ± 0.031	0.104	Dark reddish brown	Bract	Anemochory	—	Mesophyte
		*Atriplex aucheri* Moq.	AH	Utricle	304.26 ± 10.19	8.148 ± 0.291 6.428 ± 0.266 0.884 ± 0.042	0.150	Light yellowish brown	Bract	Anemochory	Ant	Mesophyte
		*Atriplex tatarica* L.	AH	Utricle	109.94 ± 3.21	4.472 ± 0.355 3.984 ± 0.302 0.566 ± 0.138	0.168	Yellowish brown	Bract	Anemochory	Ant	Mesophyte
		*Bassia dasyphylla* (Fisch. et Mey.) O. Kuntze	AH	Utricle	66.32 ± 0.78	3.018 ± 0.145 2.692 ± 0.115 0.444 ± 0.021	0.150	Light yellowish brown	Spine	Zoochory	Ant	Xerophyte
		*Bassia Sedoides* (Pall.) O. Kuntze	AH	Utricle	42.98 ± 0.49	2.958 ± 0.165 2.216 ± 0.157 1.710 ± 0.162	0.040	Yellowish brown	Hook/spine	Zoochory	—	Xerophyte
		*Camphorosma monspeliaca* L.	SS	Utricle	53.58 ± 1.45	2.238 ± 0.066 1.350 ± 0.039 0.708 ± 0.034	0.083	Yellowish brown	Hair	Anemochory	Ant	Xerophyte
		*Ceratocarpus arenarius* L.	AH	Utricle	151.14 ± 2.49	7.624 ± 0.556 10.484 ± 1.246 0.534 ± 0.028	0.364	Dark green/pale yellow	Spine	Zoochory	Ant	Mesophyte
		*Ceratoides ewersmanniana* (Stschel. ex Losinsk.) Botsch. et Ikonn.	S	Utricle	330.64 ± 4.65	7.272 ± 0.566 8.794 ± 0.790 3.680 ± 0.344	0.179	Brown	Hair	Anemochory	Ant	Mesophyte
		*Ceratoides lateens* (J. F. Gmel.) Reveal et Holmgren	S/SS	Utricle	434.80 ± 14.25	7.066 ± 0.371 8.018 ± 0.399 4.398 ± 0.206	0.076	Brown	Hair	Anemochory	Ant	Mesophyte
		*Chenopodium acuminatum* Willd.	AH	Seed	35.22 ± 0.16	0.964 ± 0.025 0.868 ± 0.024 0.416 ± 0.021	0.065	Black	None	Barochory	Ant	Mesophyte
		*Chenopodium aristatum* Linn.	AH	Seed	10.66 ± 0.22	0.594 ± 0.023 0.548 ± 0.024 0.340 ± 0.018	0.037	Black	None	Barochory	Ant	Mesophyte
		*Chenopodium glaucum* Linn.	AH	Seed	21.58 ± 0.21	0.864 ± 0.049 0.798 ± 0.051 0.342 ± 0.013	0.074	Black	None	Barochory	Ant	Mesophyte
		*Corispermum lehmannianum* Bunge.	AH	Utricle	73.10 ± 1.01	2.634 ± 0.083 1.708 ± 0.077 0.266 ± 0.016	0.143	Yellowish green	Beak	Zoochory	Ant	Mesophyte
		*Halogeton arachnoideus* Moq.	AH	Seed	43.88 ± 0.92	1.346 ± 0.061 1.102 ± 0.045 0.390 ± 0.017	0.095	Dark brown	Bract	Anemochory	Ant	Mesophyte
		*Halogeton glomeratus* (Bieb.) C. A. Mey.	AH	Seed	105.98 ± 1.12	2.092 ± 0.047 1.388 ± 0.034 0.604 ± 0.015	0.088	Yellowish brown	Bract	Anemochory	Ant	Mesophyte
		*Halostachys caspica* (Bieb.) C. A. Mey.	S	Utricle	24.24 ± 0.74	1.798 ± 0.077 1.366 ± 0.060 0.920 ± 0.057	0.046	Brown	None	Anemochory	—	Mesophyte
		*Haloxylon ammodendron* (C. A. M.) Bge.	SA	Utricle	388.80 ± 11.50	10.676 ± 0.419 9.548 ± 0.389 0.986 ± 0.091	0.172	Yellowish brown	Bract	Anemochory	Ant	Mesophyte
		*Haloxylon persicum* Bge. ex Boiss. et Buhse	SA	Utricle	797.18 ± 8.45	9.528 ± 0.149 8.820 ± 0.122 1.608 ± 0.119	0.147	Light yellowish brown	Bract	Anemochory	Ant	Mesophyte
		*Horaninowia ulicina* Fisch. et Mey.	AH	Utricle	23.00 ± 0.57	0.932 ± 0.043 0.830 ± 0.043 0.332 ± 0.014	0.083	Pale yellow	Bract	Zoochory	—	Mesophyte
		*Kalidium capsicum* (L.) Ung.-Sternb.	SS	Utricle	15.12 ± 0.20	0.728 ± 0.029 0.642 ± 0.026 0.452 ± 0.022	0.032	Light yellowish brown	None	Anemochory	—	Mesophyte
		*Kalidium cuspidatum* (Ung.-Sternb.) Grub.	SS	Utricle	11.90 ± 0.38	1.680 ± 0.127 1.336 ± 0.029 0.608 ± 0.040	0.081	Light yellowish brown	None	Anemochory	—	Mesophyte
		*Kalidium foliatum* (Pall.) G Moq.	SS	Utricle	16.06 ± 0.65	1.270 ± 0.121 0.938 ± 0.141 0.770 ± 0.081	0.047	Light yellowish brown	None	Anemochory	—	Mesophyte
		*Kochia iranica* Litv. ex Bornm.	AH	Utricle	47.34 ± 0.80	1.568 ± 0.080 1.188 ± 0.044 0.910 ± 0.050	0.037	Dark brown	Hair	Anemochory	Ant	Mesophyte
		*Petrosmonia sibirica* (Pall.) Bge.	AH	Utricle	174.96 ± 1.51	3.712 ± 0.213 1.988 ± 0.030 0.874 ± 0.039	0.104	Pale yellow	Bract	Anemochory	Ant	Mesophyte
		*Salicornia europaea* Linn.	AH	Utricle	5.54 ± 0.04	0.698 ± 0.017 0.430 ± 0.017 0.350 ± 0.017	0.049	Dark brown	None	Anemochory	—	Xerophyte
		*Salsola affinis* C. A. Mey.	AH	Utricle	626.96 ± 15.51	8.032 ± 0.408 7.106 ± 0.347 3.174 ± 0.140	0.072	Yellowish brown	Bract	Anemochory	Ant	Xerophyte
		*Salsola foliosa* (L.) Schrad.	AH	Utricle	103.94 ± 0.40	6.162 ± 0.218 5.834 ± 0.214 0.524 ± 0.020	0.182	Reddish brown	Bract	Anemochory	—	Xerophyte
		*Salsola heptapotamica* Iljin	AH	Utricle	518.34 ± 6.87	10.176 ± 0.596 9.210 ± 0.525 1.874 ± 0.183	0.139	Yellowish brown	Bract	Anemochory	Ant	Xerophyte
		*Salsola nitraria* Pall.	AH	Utricle	180.20 ± 3.96	6.690 ± 0.269 5.958 ± 0.290 1.668 ± 0.126	0.115	Yellowish brown	Bract	Anemochory	Ant	Xerophyte
		*Salsola ruthenica* Iljin	AH	Utricle	271.46 ± 4.11	8.216 ± 0.424 7.556 ± 0.397 1.748 ± 0.109	0.129	Brown	Bract	Anemochory	Ant	Xerophyte
		*Salsola subcrassa* M. Pop.	AH	Utricle	1071.36 ± 2.37	10.770 ± 0.578 9.694 ± 0.509 2.584 ± 0.120	0.117	Yellowish brown	Bract	Anemochory	Ant	Xerophyte
		*Suaeda acuminata* (C. A. Mey.) Moq.	AH	Utricle	56.88 ± 1.67	1.456 ± 0.088 1.264 ± 0.071 1.020 ± 0.056	0.020	Dark brown	None	Barochory	Ant	Xerophyte
		*Suaeda altissima* (L.) Pall.	AH	Utricle	34.74 ± 0.57	1.244 ± 0.033 1.076 ± 0.046 0.768 ± 0.033	0.029	Black	None	Barochory	Ant	Xerophyte
		*Suaeda corniculata* (C. A. Mey.) Bunge	AH	Utricle	33.79 ± 0.58	1.102 ± 0.026 0.924 ± 0.026 0.580 ± 0.021	0.047	Yellowish brown	None	Barochory	Ant	Xerophyte
		*Suaeda microphylla* (C. A. M.) Pall.	SS	Utricle	31.66 ± 0.51	1.086 ± 0.040 1.040 ± 0.039 0.964 ± 0.033	0.003	Black/yellowish brown	None	Barochory	Ant	Xerophyte
		*Suaeda physophora* Pall.	SS	Utricle	247.52 ± 2.11	2.934 ± 0.162 2.724 ± 0.120 1.870 ± 0.088	0.029	Reddish brown	Perianth	Anemochory	Ant	Mesophyte
		*Suaeda salsa* (L.) Pall.	AH	Utricle	15.20 ± 0.28	0.792 ± 0.031 0.726 ± 0.036 0.466 ± 0.020	0.034	Black	None	Barochory	Ant	Mesophyte
	Polygonaceae	*Atraphaxis frutescens* (Rgl.) Krassn.	S	Achene	283.68 ± 6.26	7.596 ± 0.096 5.322 ± 0.174 3.326 ± 0.226	0.058	Brown	Perianth	Anemochory	Ant	Xerophyte
		*Calligonum ebinuricum* Ivanova.	S	Achene	2672.20 ± 121.39	10.138 ± 0.369 6.614 ± 0.521 5.738 ± 0.437	0.045	Brown	Hook/spine	Zoochory	—	Mesophyte
		*Calligonum mongolicum* Turcz.	SS	Achene	5934.84 ± 57.75	14.056 ± 0.538 12.604 ± 0.390 12.454 ± 0.398	0.005	Yellowish brown	Hook/spine	Zoochory	—	Xerophyte
		*Calligonum leucocladum* (Schrenk) Bunge	S	Achene	2829.50 ± 124.68	10.614 ± 0.423 10.250 ± 1.378 8.240 ± 1.235	0.087	Yellowish brown	Wing	Zoochory	—	Xerophyte
		*Rumex pseudonatronatus* Borb.	PH	Achene	211.74 ± 4.07	4.614 ± 0.229 4.548 ± 0.276 3.600 ± 0.180	0.018	Yellowish brown	Bract	Anemochory	Ant	Mesophyte
	Tamaricaceae	*Reaumuria soongorica* (Pall.) Maxim.	SS	Capsule	916.18 ± 12.07	6.050 ± 0.156 2.568 ± 0.064 2.500 ± 0.058	0.078	Dark brown	Hair	Anemochory	Ant	Mesophyte
	Plumbaginaceae	*Limonium coralloides* (Tausch) Lincz.	PH	Utricle	26.40 ± 0.63	2.622 ± 0.048 1.424 ± 0.049 1.390 ± 0.050	0.051	Light brown	Bract	Anemochory	Ant	Mesophyte
		*Limonium gmelinii* (Willd.) Kuntze.	PH	Utricle	45.50 ± 1.22	3.532 ± 0.090 1.324 ± 0.062 1.288 ± 0.063	0.093	Dark brown	Bract	Anemochory	Ant	Mesophyte
		*Limonium otolepis* (Schrenk)	PH	Utricle	21.40 ± 0.42	1.984 ± 0.088 0.972 ± 0.023 0.944 ± 0.020	0.064	Light brown	Bract	Anemochory	Ant	Mesophyte
		*Limonium suffruticosum* (L.) Kuntze.	SS	Utricle	30.26 ± 0.52	2.784 ± 0.157 0.896 ± 0.066 0.868 ± 0.067	0.109	Light brown	Bract	Anemochory	Ant	Mesophyte

Rosids	Geraniaceae	*Erodium oxyrrhynchum* M. B. Fl.	AE	Capsule	225.44 ± 2.55	5.366 ± 0.119 1.086 ± 0.092 0.902 ± 0.014	0.154	Brown	Pappus/beak	Anemochory	Ant	Xerophyte

Eurosids I	Fabaceae	*Alhagi sparsifolia* Shap.	SS	Pod	487.46 ± 6.42	3.672 ± 0.129 2.292 ± 0.073 1.230 ± 0.079	0.078	Brown	None	Autochory	Ant	Xerophyte
		*Amorpha fruticosa* L.	S/SS	Pod	911.84 ± 9.71	8.616 ± 0.251 2.836 ± 0.050 1.628 ± 0.056	0.130	Nut-brown	None	Barochory	Ant	Xerophyte
		*Eremosparton songoricum* (Litv) Vass	SS	Pod	1547.34 ± 26.08	4.402 ± 0.129 3.358 ± 0.067 1.266 ± 0.044	0.091	Light yellowish brown	Awn, papery calyx	Anemochory	Ant	Mesophyte
		*Glycyrrhiza inflata* Batal.	PH	Pod	3790.04 ± 53.64	11.424 ± 0.639 4.920 ± 0.250 3.688 ± 0.141	0.091	Brown	None	Autochory	Ant	Xerophyte
		*Glycyrrhiza uralensis* Fisch	PH	Seed	903.80 ± 5.98	2.796 ± 0.151 2.301 ± 0.061 1.534 ± 0.038	0.038	Brown	None	Autochory	Ant	Mesophyte
		*Sophora alopecuroides* L.	PH	Seed	1899.66 ± 19.49	3.968 ± 0.074 2.936 ± 0.078 2.150 ± 0.081	0.039	Light brown	None	Barochory	—	Xerophyte
		*Trigonella arcuata* C. A. M.	AE	Seed	100.78 ± 1.76	2.248 ± 0.059 0.832 ± 0.028 0.656 ± 0.028	0.105	Yellowish green	None	Autochory	Ant	Xerophyte
		*Trigonella cancellata* Desf.	AE	Seed	87.84 ± 0.96	2.100 ± 0.030 0.768 ± 0.037 0.596 ± 0.030	0.107	Yellowish green	None	Autochory	Ant	Xerophyte
	Zygophyllaceae	*Nitraria roborowskii* Kom.	S	Berry	4599.56 ± 91.07	7.916 ± 0.174 4.598 ± 0.281 3.616 ± 0.137	0.059	Dark reddish brown	None	Zoochory	Ant	Xerophyte
		*Nitraria sibirica* Pall.	S	Berry	3826.70 ± 59.66	6.758 ± 0.130 3.532 ± 0.089 3.384 ± 0.084	0.055	Dark reddish brown	None	Zoochory	Ant	Xerophyte
		*Peganum harmala* Linn.	PH	Seed	289.36 ± 2.84	3.360 ± 0.046 1.832 ± 0.079 0.908 ± 0.041	0.096	Dark brown	None	Barochory	Ant	Mesophyte
		*Zygophyllum fabago* L.	PH	Seed	297.76 ± 9.15	3.704 ± 0.045 1.674 ± 0.056 0.634 ± 0.032	0.123	Brown	Balloon	Barochory	Ant	Xerophyte
		*Zygophyllum pterocarpum* Bge.	PH	Seed	170.24 ± 4.38	3.476 ± 0.089 1.884 ± 0.056 0.360 ± 0.016	0.140	Brown	Wart	Barochory	Ant	Xerophyte
		*Zygophyllum xanthoxylon* Maxim.	S	Capsule	10652.78 ± 292.29	28.588 ± 1.133 26.336 ± 1.051 15.058 ± 1.390	0.051	Pale yellow	Wing	Anemochory	Ant	Xerophyte
	Rosaceae	*Agrimonia asiatica* Juz.	PH	Achenecetum	463.34 ± 9.21	5.456 ± 0.149 3.316 ± 0.161 3.012 ± 0.194	0.044	Green	Hook/spine	Zoochory	Ant	Xerophyte
	Cannabaceae	*Cannabis sativa* L.	AH	Capsule	433.82 ± 46.22	3.016 ± 0.089 2.284 ± 0.085 1.660 ± 0.042	0.036	Grey	None	Barochory	—	Mesophyte

Eurosids II	Brassicaceae	*Alyssum deserorum* Stapf.	AE	Seed	37.32 ± 0.50	1.474 ± 0.043 1.044 ± 0.044 0.334 ± 0.013	0.107	Yellow	None	Ombro-hydrochory	—	Mesophyte
		*Alyssum linifolium* Steph. ex Willd.	AE	Seed	18.00 ± 0.29	1.330 ± 0.030 0.902 ± 0.033 0.274 ± 0.018	0.111	Yellow	None	Ombro-hydrochory	—	Xerophyte
		*Camelina microcarpa* Andrz.	AH	Seed	31.66 ± 0.24	1.164 ± 0.039 0.702 ± 0.033 0.492 ± 0.024	0.062	Yellowish brown	None	Ombro-hydrochory	—	Xerophyte
		*Camelina sativa* (Linn.) Crantz	AH	Seed	31.90 ± 0.83	1.114 ± 0.028 0.614 ± 0.019 0.502 ± 0.024	0.061	Yellowish brown	None	Ombro-hydrochory	—	Mesophyte
		*Cardaria draba* (L.) Desv.	PH	Seed	206.44 ± 0.68	2.104 ± 0.035 1.274 ± 0.059 0.828 ± 0.028	0.068	Reddish brown	None	Ombro-hydrochory	Ant	Mesophyte
		*Cardaria pubescens* (C. A. Meyer) Jarmoenko	PH	Seed	94.80 ± 1.30	1.670 ± 0.046 1.050 ± 0.027 0.620 ± 0.021	0.070	Reddish brown	None	Ombro-hydrochory	Ant	Xerophyte
		*Descurainia Sophia* (L.) Webb. ex Prantl	AH	Seed	10.60 ± 0.07	0.916 ± 0.025 0.422 ± 0.011 0.336 ± 0.013	0.081	Light reddish brown	None	Ombro-hydrochory	Ant	Xerophyte
		*Erysimum hieracifolium* L.	BPH	Seed	29.22 ± 0.34	1.324 ± 0.063 0.620 ± 0.025 0.430 ± 0.020	0.091	Brown	None	Ombro-hydrochory	Ant	Xerophyte
		*Euclidium syricum* (L.) R. Br.	AE	Silicle	400.92 ± 6.52	3.740 ± 0.163 1.898 ± 0.051 1.630 ± 0.040	0.066	Brownish green/brown	Beak	Zoochory	Ant	Xerophyte
		*Isatis* costata C. A. Mey.	BH	Silicle	394.58 ± 24.67	8.708 ± 0.430 3.904 ± 0.299 1.332 ± 0.079	0.129	Yellowish brown	Wing	Barochory	Ant	Mesophyte
		*Isatis violascens* Bge.	AE	Silicle	223.52 ± 3.15	3.038 ± 0.115 1.220 ± 0.028 0.972 ± 0.024	0.095	Yellowish brown	Wing	Barochory	Ant	Mesophyte
		*Lepidium apetalum* Willd.	ABH	Seed	18.00 ± 0.13	1.016 ± 0.023 0.620 ± 0.022 0.362 ± 0.020	0.074	Reddish brown	None	Ombro-hydrochory	Ant	Mesophyte
		*Lepidium ferganense* Korsh.	PH	Seed	21.34 ± 0.52	1.360 ± 0.022 0.684 ± 0.022 0.312 ± 0.015	0.106	Reddish brown	None	Ombro-hydrochory	—	Mesophyte
		*Lepidium latifolium* var. *affine* C. A. Mey	PH	Seed	15.22 ± 0.36	0.930 ± 0.026 0.544 ± 0.023 0.336 ± 0.015	0.074	Reddish brown	None	Ombro-hydrochory	—	Mesophyte
		*Lepidium perfoliatum* L.	ABE	Seed	78.32 ± 0.26	1.878 ± 0.042 1.188 ± 0.041 0.446 ± 0.015	0.102	Yellowish brown	None	Ombro-hydrochory	Ant	Mesophyte
		*Malcolmia africana* (L.) R. Br.	BE	Seed	13.64 ± 0.00	1.024 ± 0.040 0.567 ± 0.038 0.360 ± 0.017	0.080	Light yellowish brown	None	Barochory	Ant	Mesophyte
		*Neotorularia korolkovii* (Rgl. et Schmlh.) Hedge et J. Leonard.	ABE	Seed	9.46 ± 0.11	0.966 ± 0.037 0.448 ± 0.026 0.200 ± 0.026	0.117	Yellowish brown	None	Ombro-hydrochory	—	Mesophyte
		*Syrenia siliculosa* (M. Bieb.) Andrz.	BH	Seed	20.94 ± 0.39	1.588 ± 0.090 0.678 ± 0.026 0.420 ± 0.015	0.103	Orange	None	Ombro-hydrochory	—	Xerophyte
		*Tetracme quadricornis* (Steph.) Bge.	AE	Seed	8.00 ± 0.11	0.880 ± 0.024 0.458 ± 0.011 0.284 ± 0.016	0.086	Light yellowish brown	None	Ombro-hydrochory	—	Mesophyte
		*Thlaspi arvense* L.	AE	Seed	74.26 ± 0.46	1.598 ± 0.034 1.102 ± 0.016 0.592 ± 0.022	0.070	Dark brown	None	Barochory	Ant	Mesophyte
	Malvaceae	*Abutilon theophrasti* Medicus	AH	Seed	858.18 ± 6.37	3.396 ± 0.063 2.756 ± 0.035 1.544 ± 0.029	0.054	Dark brown	Hair	Barochory	Ant	Mesophyte
		*Althaea officinalis* L.	PH	Schizocarp	153.88 ± 2.99	2.756 ± 0.053 2.352 ± 0.042 1.292 ± 0.154	0.062	Light brown	Hair	Barochory	Ant	Xerophyte
		*Althaea nudiflora* Lindl.	BH	Schizocarp	593.78 ± 8.71	4.732 ± 0.120 4.004 ± 0.046 1.168 ± 0.069	0.111	Light brown	Hair	Barochory	Ant	Xerophyte
		*Hibiscus trionum* L.	AH	Seed	454.08 ± 5.71	2.276 ± 0.039 2.032 ± 0.036 1.392 ± 0.027	0.028	Black	Wart	Barochory	Ant	Xerophyte

Euasterids I	Scrophulariaceae	*Dodartia orientalis* L.	PH	Seed	8.72 ± 0.15	0.516 ± 0.009 0.356 ± 0.008 0.324 ± 0.009	0.028	Black	None	Barochory	—	Mesophyte
		*Leptorhabdos parviflora* Benth.	AH	Seed	111.26 ± 1.11	2.540 ± 0.103 1.096 ± 0.064 0.588 ± 0.024	0.111	Dark brown	None	Barochory	—	Mesophyte
		*Veronica ferganiea* M Pop.	AE	Seed	40.60 ± 0.65	1.458 ± 0.042 0.722 ± 0.029 0.522 ± 0.029	0.081	Brown	None	Barochory	Ant	Mesophyte
	Solanaceae	*Datura stramonium* L.	SS	Seed	691.98 ± 5.76	3.304 ± 0.046 2.608 ± 0.051 1.316 ± 0.040	0.065	Black	None	Barochory	—	Xerophyte
		*Hyoscyamus niger* L.	BH	Seed	62.94 ± 0.97	1.294 ± 0.027 1.086 ± 0.035 0.594 ± 0.025	0.057	Yellowish brown	None	Barochory	—	Xerophyte
	Boraginaceae	*Arnebia decumbens* (Vent.) Coss. et Kral.	AE	Nutlet	1335.44 ± 11.70	10.690 ± 0.345 6.384 ± 0.332 5.360 ± 0.164	0.052	Brown	Hook/spine	Zoochory	—	Xerophyte
		*Heliotropium ellipticum* Ldb.	PH	Schizocarp	95.82 ± 1.27	2.022 ± 0.145 1.260 ± 0.077 0.998 ± 0.055	0.049	Brownish green	Wart	Barochory	Ant	Xerophyte
		*Lappula myosotis* Moench	ABE	Nutlet	435.18 ± 2.71	4.354 ± 0.144 3.432 ± 0.150 3.154 ± 0.146	0.021	Dark brown	Spine	Zoochory	Ant	Xerophyte
		*Lappula spinocarpa* (Forssk.) Aschers. ex Kuntze	AE	Nutlet	1491.06 ± 57.20	3.924 ± 0.065 3.118 ± 0.037 4.112 ± 0.139	0.016	Light yellowish brown	Spine	Zoochory	Ant	Xerophyte
		*Lepechiniella lasiocarpa* W. T. Wang	AH	Nutlet	451.48 ± 4.14	2.860 ± 0.107 2.778 ± 0.101 2.606 ± 0.126	0.015	Brown	Spine	Zoochory	Ant	Xerophyte
		*Lindelofia stylosa* (Kar. et Kir.) Brand.	PH	Nutlet	1397.80 ± 28.88	6.768 ± 0.069 4.648 ± 0.083 2.248 ± 0.044	0.078	Yellowish brown	Spine	Zoochory	Ant	Mesophyte
	Lamiaceae	*Elsholtzia densa* Benth.	PH	Nutlet	10.02 ± 0.09	0.688 ± 0.010 0.508 ± 0.020 0.468 ± 0.018	0.023	Dark brown	Wart	Barochory	Ant	Xerophyte
		*Leonurus turkestanicus* V. Krecz. et Rupr.	PH	Nutlet	111.60 ± 0.56	2.364 ± 0.038 1.396 ± 0.060 0.720 ± 0.046	0.086	Light brown	None	Barochory	Ant	Mesophyte
		*Marrubium vulgare* L.	PH	Nutlet	99.34 ± 0.55	1.784 ± 0.055 1.060 ± 0.038 0.724 ± 0.015	0.066	Dark brown	Wart	Barochory	Ant	Mesophyte
		*Phlomis chinghoensis* C. Y. Wu	PH	Nutlet	556.84 ± 8.71	4.268 ± 0.083 2.380 ± 0.074 1.280 ± 0.340	0.087	Dark brown	None	Barochory	Ant	Mesophyte
		*Salvia deserta* Schang.	PH	Nutlet	51.58 ± 1.24	1.576 ± 0.030 1.164 ± 0.033 0.700 ± 0.025	0.055	Black	None	Barochory	Ant	Mesophyte
	Plantaginaceae	*Plantago lessingii* Fisch. et Mey.	AE	Seed	193.74 ± 2.13	3.514 ± 0.064 1.328 ± 0.059 0.610 ± 0.041	0.129	Yellowish brown	None	Ombro-hydrochory	Ant	Mesophyte
		*Plantago major* L.	PH	Seed	17.66 ± 0.38	1.132 ± 0.049 0.656 ± 0.015 0.372 ± 0.016	0.080	Yellowish brown	None	Ombro-hydrochory	Ant	Xerophyte
		*Plantago maritima* Linn. subsp. *ciliata* Printz.	PH	Seed	44.06 ± 0.57	1.756 ± 0.054 0.824 ± 0.044 0.426 ± 0.023	0.105	Yellowish brown	None	Ombro-hydrochory	Ant	Xerophyte
		*Plantago minuta* Pall.	AE	Seed	203.80 ± 5.43	3.534 ± 0.090 1.366 ± 0.033 0.586 ± 0.017	0.129	Dark brown	None	Ombro-hydrochory	Ant	Xerophyte
	Convolvulaceae	*Cuscuta australis* R. Br.	AH	Seed	77.36 ± 3.33	1.260 ± 0.048 1.028 ± 0.038 0.732 ± 0.026	0.031	Light brown	None	Barochory	Ant	Mesophyte
	Rubiaceae	*Galium rivale* (Sibth. et Smith) Griseb.	PH	Seed	35.44 ± 0.13	1.056 ± 0.031 0.728 ± 0.022 0.592 ± 0.037	0.041	Dark brown	Wart	Barochory	Ant	Xerophyte

Euasterids II	Campanulaceae	*Codonopsis clematidea* (Schrenk) C. B. Clarke	PH	Seed	50.18 ± 0.41	1.384 ± 0.036 0.636 ± 0.027 0.584 ± 0.027	0.074	Light brown	None	Barochory	Ant	Mesophyte
	Asteraceae	*Arctium lappa* L.	BH	Achene	1153.06 ± 9.95	6.272 ± 0.063 2.556 ± 0.089 1.320 ± 0.022	0.117	Brown	Pappus	Anemochory	Ant	Mesophyte
		*Arctium tomentosum* Mill.	BH	Achene	931.96 ± 13.94	5.412 ± 0.028 2.376 ± 0.076 1.324 ± 0.030	0.107	Brown	Pappus	Anemochory	Ant	Mesophyte
		*Artemisia annua* L.	AH	Achene	5.00 ± 0.14	1.208 ± 0.016 0.384 ± 0.017 0.308 ± 0.021	0.119	Light brown	None	Anemochory	Ant	Mesophyte
		*Artemisia ordosica* Krasch	SS	Achenecetum	245.66 ± 4.02	1.546 ± 0.033 0.572 ± 0.022 0.370 ± 0.014	0.115	Brown	None	Anemochory	Ant	Mesophyte
		*Cancrinia discoidea* (Ledeb.) Poljak.	BH	Achene	21.74 ± 0.29	2.932 ± 0.175 0.908 ± 0.027 0.878 ± 0.022	0.111	Pale yellow	Pappus	Anemochory	—	Xerophyte
		*Centaurea squarosa* Willd.	BPH	Achenecetum	1860.14 ± 30.25	9.638 ± 0.294 5.160 ± 0.087 5.016 ± 0.137	0.053	Pale yellow	Hook/spine	Zoochory	Ant	Xerophyte
		*Cichorium intybus* L.	PH	Achene	93.22 ± 2.10	2.988 ± 0.044 1.140 ± 0.065 0.552 ± 0.044	0.127	Yellowish brown	Pappus	Anemochory	Ant	Mesophyte
		*Cousinia affinis* Schrenk	PH	Achene	424.78 ± 4.07	4.876 ± 0.196 2.560 ± 0.077 1.384 ± 0.052	0.093	Greyish black	Hook/spine	Zoochory	Ant	Mesophyte
		*Garhadiolus papposus* Boiss. et Buhse	AE	Achene	230.00 ± 2.94	5.904 ± 0.205 3.158 ± 0.113 1.114 ± 0.076	0.116	Light yellowish brown	Pappus/beak	Anemochory	Ant	Mesophyte
		*Koelpinia linearis* Pall.	AE	Achene	552.36 ± 3.95	10.262 ± 0.329 6.682 ± 0.293 2.244 ± 0.217	0.111	Brown	Hook/spine	Zoochory	Ant	Xerophyte
		*Neopallasia pectinata* (Pall.) Poljak.	AH	Achene	33.70 ± 1.83	1.628 ± 0.047 0.758 ± 0.021 0.320 ± 0.012	0.116	Dark brown	None	Anemochory	—	Mesophyte
		*Onopordum acanthium* L.	BH	Achene	982.10 ± 7.56	4.852 ± 0.053 2.556 ± 0.039 1.410 ± 0.034	0.090	Grey/greyish black	None	Barochory	Ant	Mesophyte
		*Saussurea salsa* (Pall.) Spreng.	PH	Achene	129.22 ± 4.70	3.522 ± 0.134 1.302 ± 0.053 0.712 ± 0.047	0.123	Greyish white	Pappus	Anemochory	Ant	Mesophyte
		*Xanthium mongolicum* Kitag.	AH	Achene	19317.88 ± 131.28	22.088 ± 0.579 13.344 ± 0.219 12.596 ± 0.332	0.040	Yellowish green/green	Beak/hook/spine	Zoochory	—	Mesophyte
	Apiaceae	*Conium maculatum* L.	BH	Schizocarp	278.92 ± 8.04	2.852 ± 0.069 1.540 ± 0.013 1.104 ± 0.044	0.071	Light yellowish brown	None	Barochory	—	Mesophyte
		*Soranthus meyeri* Ledeb.	PH	Schizocarp	189.68 ± 1.07	4.492 ± 0.156 3.366 ± 0.158 0.806 ± 0.070	0.130	Pale yellow	None	Barochory	Ant	Xerophyte

Notes: AH: annuals; ABH: annuals/biennials; BH: biennials; BPH: biennials/perennials; PH: perennials; S: shrubs; SS: semishrubs; SA: small arbor; AE: annuals ephemerals; ABE: annuals/biennials ephemerals; BE: biennial ephemerals.

**Table 2:** Multiway tests of between-subjects effects.

Source	Seed mass				Seed shape		
	df	*F*	Sig.	*R* ^2^	*F*	Sig.	*R* ^2^
Model	20	5.833	0.000	0.481	2.725	0.000	0.302
APG	7	5.856	0.000	0.169	3.185	0.004	0.125
Vegetative period	8	2.152	0.036	0.071	1.223	0.291	0.053
Dispersal syndromes	4	9.733	0.000	0.160	1.748	0.143	0.040
Ecotype	1	0.248	0.619	0.001	0.313	0.577	0.003

*Remove APG *							
Model	13	4.654	0.000	0.309	2.168	0.014	0.173
Vegetative period	8	2.853	0.006	0.117	1.509	0.160	0.073
Dispersal syndromes	4	7.650	0.000	0.157	4.706	0.001	0.115
Ecotype	1	1.490	0.224	0.008	0.584	0.446	0.003

*Remove vegetative period *							
Model	12	7.974	0.000	0.415	3.686	0.000	0.247
APG	7	7.094	0.000	0.215	3.782	0.001	0.149
Dispersal syndromes	4	11.135	0.000	0.193	1.296	0.275	0.029
Ecotype	1	0.432	0.512	0.002	0.390	0.533	0.003

*Remove dispersal syndromes *							
Model	16	3.829	0.000	0.320	2.902	0.000	0.263
APG	7	4.444	0.000	0.163	5.068	0.000	0.202
Vegetative period	8	2.474	0.016	0.103	1.002	0.438	0.045
Ecotype	1	0.944	0.333	0.005	0.255	0.614	0.003

*Remove ecotype *							
Model	19	6.164	0.000	0.480	2.867	0.000	0.300
APG	7	6.103	0.000	0.175	3.322	0.003	0.127
Vegetative period	8	2.184	0.033	0.072	1.246	0.278	0.056
Dispersal syndromes	4	10.030	0.000	0.164	1.745	0.144	0.037
